# 12-Acetyl-6-hy­droxy-3,3,9,9-tetra­methyl­furo[3,4-*b*]pyrano[3,2-*h*]xanthene-7,11(3*H*,9*H*)-dione

**DOI:** 10.1107/S1600536810048592

**Published:** 2010-11-27

**Authors:** Gwendoline Cheng Lian Ee, Siow Hwa Teo, Huey Chong Kwong, Mohamed Ibrahim Mohamed Tahir, Sidik Silong

**Affiliations:** a Department of Chemistry, Faculty of Science, University Putra Malaysia, 43400 UPM Serdang, Selangor, Malaysia

## Abstract

The title compound, Artonol B, C_24_H_20_O_7_, isolated from the stem bark of *Artocarpus kemando*, consists of four six-membered rings and one five-membered ring. The tricyclic xanthone ring system is almost planar [maximum deviation 0.115 (5) Å], whereas the pyran­oid ring is in a distorted boat conformation·The furan ring is almost coplanar with the fused aromatic ring, making a dihedral angle of 3.76 (9)°. The phenol ring serves as a intra­molecular hydrogen-bond donor to the adjacent carbonyl group and also acts as an inter­molecular hydrogen-bond acceptor for the methyl groups of adjacent mol­ecules, forming a three-dimensional network in the crystal.

## Related literature

For bond-length data, see Allen *et al.* (1987 ▶). For related structures, see: Doriguetto *et al.* (2001 ▶); Marek *et al.* (2003 ▶); Boonnak *et al.* (2007 ▶). For the biological activity of flavonoids from *Artocarpus kemando* and other species of *Artocarpus*, see: Burkill (1935 ▶); Makmur *et al.* (1999 ▶); Wei *et al.* (2005 ▶); Toshio *et al.* (2003 ▶); Lin *et al.* (1996 ▶); Shimizu *et al.* (2000 ▶); Patil *et al.* (2002 ▶); Tati *et al.* (2001 ▶). For a description of the Cambridge Structural Database, see: Allen (2002 ▶).
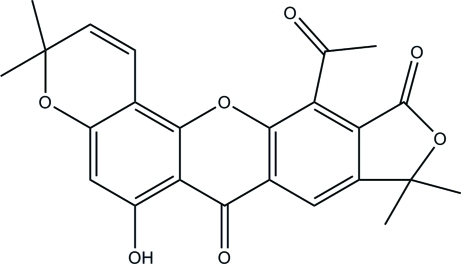


## Experimental

### 

#### Crystal data

C_24_H_20_O_7_
*M* *_r_* = 420.42
Monoclinic, 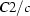

*a* = 36.511 (2) Å
*b* = 5.3275 (2) Å
*c* = 20.0218 (8) Å
β = 96.318 (5)°
*V* = 3870.8 (3) Å^3^
*Z* = 8
Cu *K*α radiation
μ = 0.89 mm^−1^
*T* = 150 K
0.30 × 0.28 × 0.04 mm

#### Data collection

Oxford Diffraction Gemini E diffractometer
Absorption correction: multi-scan (*CrysAlis RED*; Oxford Diffraction, 2006 ▶) *T*_min_ = 0.780, *T*_max_ = 0.965
17338 measured reflections
3813 independent reflections
3383 reflections with *I* > 2.0σ(*I*)
*R*_int_ = 0.018

#### Refinement

*R*[*F* ^2^ > 2σ(*F* ^2^)] = 0.039
*wR*(*F* ^2^) = 0.110
*S* = 0.99
3813 reflections
280 parameters
H-atom parameters constrained
Δρ_max_ = 0.32 e Å^−3^
Δρ_min_ = −0.21 e Å^−3^

### 

Data collection: *CrysAlis CCD* (Oxford Diffraction, 2006 ▶); cell refinement: *CrysAlis CCD*; data reduction: *CrysAlis RED* (Oxford Diffraction, 2006 ▶); program(s) used to solve structure: *SIR92* (Altomare *et al.*, 1994 ▶); program(s) used to refine structure: *CRYSTALS* (Betteridge *et al.*, 2003 ▶); molecular graphics: *Mercury* (Macrae *et al.*, 2006 ▶); software used to prepare material for publication: *CRYSTALS*.

## Supplementary Material

Crystal structure: contains datablocks global, I. DOI: 10.1107/S1600536810048592/kp2279sup1.cif

Structure factors: contains datablocks I. DOI: 10.1107/S1600536810048592/kp2279Isup2.hkl

Additional supplementary materials:  crystallographic information; 3D view; checkCIF report

## Figures and Tables

**Table 1:** Hydrogen-bond geometry (Å, °)

*D*—H⋯*A*	*D*—H	H⋯*A*	*D*⋯*A*	*D*—H⋯*A*
C16—H161⋯O15^i^	0.96	2.55	3.4062 (19)	148
C20—H202⋯O13^i^	0.97	2.53	3.4918 (19)	171
O22—H221⋯O5	0.88	1.78	2.5922 (19)	153
C31—H313⋯O22^ii^	0.97	2.57	3.5170 (19)	165
C27—H271⋯O5^iii^	0.94	2.58	3.4923 (19)	163

Symmetry codes: (i) 
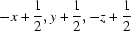
; (ii) 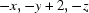; (iii) 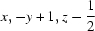.

**Fig. 1.**
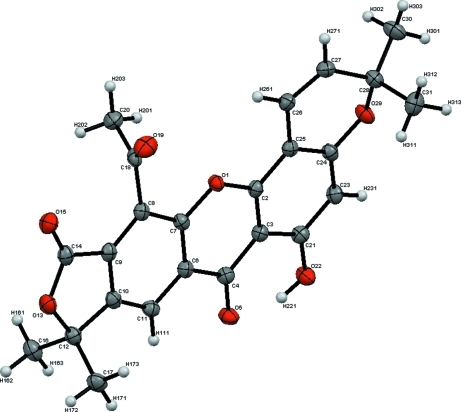
Structure and the labeling scheme for 12-acetyl-6-hydroxy-3,3,9,9-tetramethylfuro[3,4-b]pyrano[3,2-h]xanthene-7,11(3H,9H)-dione. Displacement ellipsoids are drawn at the 50% probability level.

## supplementary crystallographic information

### Comment

Flavonoids are polyphenolic compounds which are important for human health. Previous studies on flavonoids from this plant and other species of *Artocarpus* have revealed their wide range of pharmacological activities (Wei, *et al.*, 2005; Toshio, *et al.*, 2003; Lin *et al.*, 1996; Shimizu *et al.*, 2000; Patil *et al.*, 2002; Tati *et al.*, 2001). *Artocarpus kemando*, a tree of the forests and swamps is distributed in Thailand, Peninsular Malaysia, Sumatra and Borneo island (Burkill, 1935). In our continuing search for anti-cancer hit compounds we decided to look at *Artocarpus kemando.* We found that the chloroform extract of the stem bark of *Artocarpus kemando* displayed significant growth inhibition activities towards HL-60 cell lines and obtained Artonol B (I).

The molecular structure of (I) (Fig. 1) with the xanthone skeleton (ring B, C and D) is nearly planar with the exception of the atom C8 with the deviation from planarity of 0.115 Å. Rings A, B, C and D are individually almost planar, including the O5, O15 abd O22 atoms that are linked to them. The largest deviations from the individiual least-squares planes are 0.030 Å, 0.023 Å, 0.037 Å and 0.019 Å for ring A, B, C and D, respectively. Rings A and B form a dihedral angle of 3.06°, those of B and C ring form an angle of 4.23° and rings C and D form an dihedral angle of 3.42°. The planes of rings B and D intersect on a line which is approximately through the middle of ring C and gives rise to a dihedral angle of 7.65°. The mean torsion angle of ring D is 16.26° and it adopts a conformation half way between an envolope and a half-boat. The major pucking is in ring D at C28, owing to the constraint of the double bond between C26 and C27.

Bond distances and angles in the titled compound are in normal range (Allen *et al.*, 1987). The average value of C—O1 bond lengths in pyranoid ring C is 1.368 Å and the observed geometries of pyranoid ring C are comparable to other reported pyranoxanthone geometries (Doriguetto *et al.*, 2001; Marek *et al.*,2003; Boonnak *et al.*, 2007). The crystal structure is stabilised by intra- and intermolecular O—H···O and C—H···O hydrogen bonding. The titled molecules exhibit a moderate intramolecular hydrogen bond O22—H221···O5, with O···O = 2.5922 (19) Å. Meanwhile, the H atom of the C31 methyl group forms a hydrogen bond with O22 at (-*x*, -*y* + 2, -*z*) [the C···O distance is 3.5170 (19) Å]. The H atom at C27 forms a hydrogen bond with O5 at (*x*, -*y* + 1, *z* - 1/2)[the C···O distance is 3.4923 (19) Å](Table 1).

The cystallographic data of this crystal structure has been deposited at Cambridge Crystallographic Data Center with deposition number CCDC 796169 (Allen, 2002).

### Experimental

The powdered stem bark (4.7 kg) of *Artocarpus kemando* were defatted with n-hexane and sequentially extracted using methanol at room temperature for more than 48 h. This resulted in 198.5 g of methanol extract. The methanol extract was dissolved in a water-acetone mixture (1: 3, 500 mL) and the soluble portion was partitioned using chloroform (CHCl_3_) (3 × 400 mL) to afford a crude chloroform extract (20 g). Repeated silica gel column chromatographic separation on the chloroform extract (20 g) (hexane, hexane-chloroform, chloroform-ethyl acetate, ethyl acetate-methanol and methanol in order of increasing polarity) followed by radial chromatography yielded pure artonol B (I), fine yellow solid with melting point 462-467 K. Good single crystals for X-ray diffraction were prepared by slow evaporation and diffusion of diethyl ether into a solution of (I) in chloroform at room temperature.

### Refinement

The H atoms were all located in a difference map, but those attached to carbon atoms were repositioned geometrically. The H atoms were initially refined with soft restraints on the bond lengths and angles to regularize their geometry (C—H in the range 0.93-0.98, N—H in the range 0.86–0.89 N—H to 0.86 O—H = 0.82Å) and U_iso_(H) (in the range 1.2–1.5 times U_eq_ of the parent atom), after which the positions were refined with riding constraints.

### Crystal data

**Table:**

C_24_H_20_O_7_	*F*(000) = 1760
*M**_r_* = 420.42	*D*_x_ = 1.443 Mg m^−^^3^
Monoclinic, *C*2/*c*	Melting point: 189 K
Hall symbol: -C 2yc	Cu *K*α radiation, λ = 1.54184 Å
*a* = 36.511 (2) Å	Cell parameters from 8995 reflections
*b* = 5.3275 (2) Å	θ = 72.0–3.5°
*c* = 20.0218 (8) Å	µ = 0.89 mm^−^^1^
β = 96.318 (5)°	*T* = 150 K
*V* = 3870.8 (3) Å^3^	Plate, yellow
*Z* = 8	0.30 × 0.28 × 0.04 mm

### Data collection

**Table:**

Oxford Diffraction Gemini E diffractometer	3813 independent reflections
Radiation source: sealed x-ray tube	3383 reflections with *I* > 2.0σ(*I*)
graphite	*R*_int_ = 0.018
ω/2θ scans	θ_max_ = 72.1°, θ_min_ = 4.4°
Absorption correction: multi-scan (*CrysAlis RED*; Oxford Diffraction, 2006)	*h* = −44→41
*T*_min_ = 0.780, *T*_max_ = 0.965	*k* = −6→6
17338 measured reflections	*l* = −15→24

### Refinement

**Table:**

Refinement on *F*^2^	Primary atom site location: structure-invariant direct methods
Least-squares matrix: full	Hydrogen site location: inferred from neighbouring sites
*R*[*F*^2^ > 2σ(*F*^2^)] = 0.039	H-atom parameters constrained
*wR*(*F*^2^) = 0.110	*w* = 1/[σ^2^(*F*^2^) + (0.07*P*)^2^ + 3.68*P*], where *P* = [max(*F*_o_^2^,0) + 2*F*_c_^2^]/3
*S* = 0.99	(Δ/σ)_max_ = 0.0003
3813 reflections	Δρ_max_ = 0.32 e Å^−^^3^
280 parameters	Δρ_min_ = −0.21 e Å^−^^3^
0 restraints	

### Fractional atomic coordinates and isotropic or equivalent isotropic displacement parameters (Å^2^)

**Table:**

	*x*	*y*	*z*	*U*_iso_*/*U*_eq_	
O1	0.12808 (2)	0.38440 (18)	0.09348 (4)	0.0198	
C2	0.10246 (3)	0.5702 (2)	0.07942 (6)	0.0185	
C3	0.09197 (4)	0.7295 (2)	0.12975 (6)	0.0191	
C4	0.10576 (4)	0.6903 (3)	0.19901 (6)	0.0202	
O5	0.09659 (3)	0.82336 (19)	0.24578 (5)	0.0261	
C6	0.13177 (3)	0.4810 (2)	0.21237 (6)	0.0195	
C7	0.14301 (4)	0.3455 (3)	0.15819 (6)	0.0190	
C8	0.16981 (4)	0.1566 (3)	0.16701 (7)	0.0197	
C9	0.18272 (4)	0.0989 (2)	0.23308 (7)	0.0201	
C10	0.17096 (4)	0.2259 (3)	0.28733 (6)	0.0204	
C11	0.14611 (4)	0.4195 (3)	0.27803 (7)	0.0205	
H111	0.1386	0.5096	0.3144	0.0256*	
C12	0.18915 (4)	0.1142 (3)	0.35190 (7)	0.0224	
O13	0.21047 (3)	−0.09518 (18)	0.32781 (5)	0.0251	
C14	0.20880 (4)	−0.1013 (3)	0.25958 (7)	0.0223	
O15	0.22540 (3)	−0.2519 (2)	0.23027 (5)	0.0287	
C16	0.21617 (4)	0.2917 (3)	0.39064 (7)	0.0288	
H163	0.2033	0.4353	0.4063	0.0429*	
H162	0.2289	0.2059	0.4301	0.0439*	
H161	0.2342	0.3494	0.3624	0.0428*	
C17	0.16119 (4)	0.0067 (3)	0.39501 (7)	0.0277	
H171	0.1481	0.1395	0.4145	0.0409*	
H172	0.1737	−0.0925	0.4316	0.0407*	
H173	0.1438	−0.0978	0.3681	0.0410*	
C18	0.18478 (4)	0.0411 (3)	0.10628 (7)	0.0213	
O19	0.17671 (3)	−0.1689 (2)	0.08765 (6)	0.0334	
C20	0.20997 (4)	0.2106 (3)	0.07285 (7)	0.0271	
H203	0.2185	0.1277	0.0339	0.0398*	
H202	0.2306	0.2608	0.1047	0.0413*	
H201	0.1967	0.3604	0.0575	0.0414*	
C21	0.06673 (4)	0.9261 (2)	0.11022 (7)	0.0208	
O22	0.05637 (3)	1.08580 (19)	0.15665 (5)	0.0275	
H221	0.0674	1.0316	0.1953	0.0432*	
C23	0.05232 (4)	0.9535 (3)	0.04379 (7)	0.0221	
C24	0.06218 (4)	0.7823 (2)	−0.00367 (6)	0.0198	
C25	0.08781 (4)	0.5891 (2)	0.01261 (6)	0.0194	
C26	0.09776 (4)	0.4252 (3)	−0.04086 (7)	0.0215	
C27	0.07852 (4)	0.4357 (3)	−0.10100 (7)	0.0225	
C28	0.04539 (4)	0.6033 (3)	−0.11489 (6)	0.0220	
O29	0.04714 (3)	0.81622 (18)	−0.06783 (5)	0.0252	
C30	0.04382 (5)	0.7261 (3)	−0.18347 (7)	0.0318	
H301	0.0229	0.8361	−0.1902	0.0461*	
H302	0.0663	0.8214	−0.1864	0.0468*	
H303	0.0418	0.6015	−0.2176	0.0477*	
C31	0.01040 (4)	0.4563 (3)	−0.10626 (8)	0.0307	
H311	0.0115	0.3962	−0.0603	0.0455*	
H313	−0.0111	0.5621	−0.1161	0.0455*	
H312	0.0084	0.3170	−0.1366	0.0447*	
H271	0.0845	0.3337	−0.1366	0.0260*	
H261	0.1176	0.3146	−0.0317	0.0269*	
H231	0.0354	1.0844	0.0313	0.0277*	

### Atomic displacement parameters (Å^2^)

**Table:**

	*U*^11^	*U*^22^	*U*^33^	*U*^12^	*U*^13^	*U*^23^
O1	0.0208 (5)	0.0204 (5)	0.0177 (4)	0.0048 (4)	−0.0006 (3)	−0.0008 (3)
C2	0.0172 (6)	0.0169 (6)	0.0212 (6)	0.0004 (5)	0.0018 (5)	0.0013 (5)
C3	0.0189 (6)	0.0175 (6)	0.0208 (6)	−0.0001 (5)	0.0021 (5)	0.0000 (5)
C4	0.0210 (6)	0.0188 (6)	0.0209 (6)	0.0001 (5)	0.0029 (5)	−0.0002 (5)
O5	0.0325 (5)	0.0262 (5)	0.0197 (5)	0.0085 (4)	0.0024 (4)	−0.0030 (4)
C6	0.0189 (6)	0.0183 (6)	0.0212 (6)	−0.0004 (5)	0.0016 (5)	0.0001 (5)
C7	0.0196 (6)	0.0184 (6)	0.0184 (6)	−0.0014 (5)	−0.0003 (5)	0.0008 (5)
C8	0.0188 (6)	0.0177 (6)	0.0224 (6)	−0.0007 (5)	0.0012 (5)	−0.0005 (5)
C9	0.0183 (6)	0.0179 (6)	0.0239 (6)	−0.0017 (5)	0.0007 (5)	0.0016 (5)
C10	0.0197 (6)	0.0202 (6)	0.0208 (6)	−0.0036 (5)	−0.0001 (5)	0.0019 (5)
C11	0.0223 (6)	0.0197 (6)	0.0194 (6)	−0.0015 (5)	0.0024 (5)	−0.0011 (5)
C12	0.0235 (7)	0.0204 (7)	0.0225 (6)	0.0008 (5)	−0.0003 (5)	0.0025 (5)
O13	0.0259 (5)	0.0233 (5)	0.0252 (5)	0.0044 (4)	−0.0011 (4)	0.0042 (4)
C14	0.0204 (6)	0.0206 (7)	0.0254 (7)	−0.0017 (5)	−0.0008 (5)	0.0028 (5)
O15	0.0273 (5)	0.0257 (5)	0.0332 (5)	0.0074 (4)	0.0034 (4)	0.0004 (4)
C16	0.0289 (7)	0.0285 (8)	0.0274 (7)	−0.0030 (6)	−0.0047 (6)	0.0012 (6)
C17	0.0302 (7)	0.0287 (7)	0.0242 (7)	−0.0029 (6)	0.0023 (6)	0.0047 (6)
C18	0.0184 (6)	0.0217 (7)	0.0227 (6)	0.0049 (5)	−0.0027 (5)	−0.0015 (5)
O19	0.0353 (6)	0.0259 (6)	0.0398 (6)	−0.0016 (5)	0.0080 (5)	−0.0107 (5)
C20	0.0250 (7)	0.0298 (8)	0.0269 (7)	0.0021 (6)	0.0044 (5)	−0.0014 (6)
C21	0.0221 (6)	0.0178 (6)	0.0227 (6)	0.0005 (5)	0.0029 (5)	−0.0008 (5)
O22	0.0335 (5)	0.0253 (5)	0.0229 (5)	0.0117 (4)	−0.0007 (4)	−0.0034 (4)
C23	0.0225 (6)	0.0179 (6)	0.0254 (7)	0.0036 (5)	−0.0001 (5)	0.0024 (5)
C24	0.0210 (6)	0.0189 (6)	0.0189 (6)	−0.0017 (5)	−0.0001 (5)	0.0028 (5)
C25	0.0191 (6)	0.0187 (6)	0.0203 (6)	−0.0007 (5)	0.0020 (5)	0.0005 (5)
C26	0.0202 (6)	0.0229 (7)	0.0217 (6)	0.0023 (5)	0.0032 (5)	0.0002 (5)
C27	0.0241 (7)	0.0236 (7)	0.0203 (6)	−0.0002 (5)	0.0047 (5)	−0.0009 (5)
C28	0.0248 (7)	0.0223 (7)	0.0183 (6)	−0.0003 (5)	−0.0005 (5)	0.0001 (5)
O29	0.0325 (5)	0.0213 (5)	0.0203 (5)	0.0048 (4)	−0.0042 (4)	0.0008 (4)
C30	0.0414 (9)	0.0323 (8)	0.0207 (7)	0.0030 (7)	−0.0013 (6)	0.0039 (6)
C31	0.0237 (7)	0.0268 (8)	0.0415 (8)	0.0009 (6)	0.0024 (6)	0.0010 (6)

### Geometric parameters (Å, °)

**Table:**

O1—C2	1.3696 (16)	C17—H173	0.962
O1—C7	1.3651 (15)	C18—O19	1.2052 (18)
C2—C3	1.4025 (18)	C18—C20	1.4982 (19)
C2—C25	1.3886 (18)	C20—H203	0.976
C3—C4	1.4375 (18)	C20—H202	0.968
C3—C21	1.4213 (18)	C20—H201	0.967
C4—O5	1.2490 (16)	C21—O22	1.3449 (16)
C4—C6	1.4700 (18)	C21—C23	1.3833 (19)
C6—C7	1.4016 (18)	O22—H221	0.880
C6—C11	1.3996 (18)	C23—C24	1.3926 (19)
C7—C8	1.4009 (19)	C23—H231	0.947
C8—C9	1.3891 (18)	C24—C25	1.4050 (19)
C8—C18	1.5175 (18)	C24—O29	1.3524 (15)
C9—C10	1.3875 (19)	C25—C26	1.4582 (18)
C9—C14	1.4879 (18)	C26—C27	1.3269 (19)
C10—C11	1.3727 (19)	C26—H261	0.937
C10—C12	1.5093 (18)	C27—C28	1.5048 (19)
C11—H111	0.938	C27—H271	0.941
C12—O13	1.4715 (17)	C28—O29	1.4714 (16)
C12—C16	1.5166 (19)	C28—C30	1.5162 (18)
C12—C17	1.5196 (19)	C28—C31	1.524 (2)
O13—C14	1.3610 (17)	C30—H301	0.960
C14—O15	1.1973 (17)	C30—H302	0.972
C16—H163	0.969	C30—H303	0.950
C16—H162	0.984	C31—H311	0.972
C16—H161	0.962	C31—H313	0.970
C17—H171	0.961	C31—H312	0.957
C17—H172	0.975		
			
C2—O1—C7	119.81 (10)	H172—C17—H173	109.4
O1—C2—C3	121.60 (11)	C8—C18—O19	121.81 (13)
O1—C2—C25	115.63 (11)	C8—C18—C20	113.98 (11)
C3—C2—C25	122.77 (12)	O19—C18—C20	124.21 (13)
C2—C3—C4	120.74 (12)	C18—C20—H203	110.5
C2—C3—C21	117.93 (12)	C18—C20—H202	110.0
C4—C3—C21	121.32 (12)	H203—C20—H202	111.0
C3—C4—O5	123.14 (12)	C18—C20—H201	109.1
C3—C4—C6	115.85 (11)	H203—C20—H201	108.3
O5—C4—C6	121.01 (12)	H202—C20—H201	107.9
C4—C6—C7	119.24 (12)	C3—C21—O22	119.96 (12)
C4—C6—C11	121.07 (12)	C3—C21—C23	120.59 (12)
C7—C6—C11	119.68 (12)	O22—C21—C23	119.44 (12)
C6—C7—O1	122.41 (12)	C21—O22—H221	105.3
C6—C7—C8	122.17 (12)	C21—C23—C24	119.16 (12)
O1—C7—C8	115.41 (11)	C21—C23—H231	120.0
C7—C8—C9	116.00 (12)	C24—C23—H231	120.8
C7—C8—C18	119.90 (11)	C23—C24—C25	122.48 (12)
C9—C8—C18	123.96 (12)	C23—C24—O29	116.88 (12)
C8—C9—C10	122.38 (12)	C25—C24—O29	120.57 (12)
C8—C9—C14	129.44 (12)	C24—C25—C2	116.94 (12)
C10—C9—C14	108.15 (12)	C24—C25—C26	118.80 (12)
C9—C10—C11	121.15 (12)	C2—C25—C26	124.25 (12)
C9—C10—C12	109.47 (12)	C25—C26—C27	119.42 (12)
C11—C10—C12	129.38 (12)	C25—C26—H261	118.9
C6—C11—C10	118.48 (12)	C27—C26—H261	121.7
C6—C11—H111	120.0	C26—C27—C28	121.82 (12)
C10—C11—H111	121.6	C26—C27—H271	121.3
C10—C12—O13	102.52 (10)	C28—C27—H271	116.8
C10—C12—C16	113.10 (12)	C27—C28—O29	111.21 (11)
O13—C12—C16	107.61 (11)	C27—C28—C30	111.89 (12)
C10—C12—C17	112.04 (11)	O29—C28—C30	104.02 (11)
O13—C12—C17	108.25 (11)	C27—C28—C31	109.92 (12)
C16—C12—C17	112.61 (12)	O29—C28—C31	107.55 (11)
C12—O13—C14	112.35 (10)	C30—C28—C31	112.06 (12)
C9—C14—O13	107.28 (11)	C28—O29—C24	119.33 (10)
C9—C14—O15	130.08 (13)	C28—C30—H301	110.0
O13—C14—O15	122.60 (12)	C28—C30—H302	109.4
C12—C16—H163	110.2	H301—C30—H302	109.6
C12—C16—H162	110.1	C28—C30—H303	110.0
H163—C16—H162	108.0	H301—C30—H303	109.1
C12—C16—H161	110.3	H302—C30—H303	108.8
H163—C16—H161	109.0	C28—C31—H311	109.2
H162—C16—H161	109.1	C28—C31—H313	110.4
C12—C17—H171	110.4	H311—C31—H313	109.4
C12—C17—H172	110.0	C28—C31—H312	109.4
H171—C17—H172	107.9	H311—C31—H312	109.8
C12—C17—H173	110.0	H313—C31—H312	108.6
H171—C17—H173	109.1		

### Hydrogen-bond geometry (Å, °)

**Table:**

*D*—H···*A*	*D*—H	H···*A*	*D*···*A*	*D*—H···*A*
C16—H161···O15^i^	0.96	2.55	3.4062 (19)	148
C20—H202···O13^i^	0.97	2.53	3.4918 (19)	171
O22—H221···O5	0.88	1.78	2.5922 (19)	153
C31—H313···O22^ii^	0.97	2.57	3.5170 (19)	165
C27—H271···O5^iii^	0.94	2.58	3.4923 (19)	163

Symmetry codes: (i) −*x*+1/2, *y*+1/2, −*z*+1/2; (ii) −*x*, −*y*+2, −*z*; (iii) *x*, −*y*+1, *z*−1/2.
